# Plasmid-induced cytotoxicity revealed by nanopore and nanostraw electroporation

**DOI:** 10.1039/d5nr02352a

**Published:** 2025-09-08

**Authors:** Frida Ekstrand, Sara Davidsson Bencker, Sabrina Ruhrmann, Yupeng Yang, Charlotte Ling, Christelle N. Prinz

**Affiliations:** a Division of Solid State Physics, Lund University 221 00 Lund Sweden christelle.prinz@ftf.lth.se; b NanoLund, Lund University 221 00 Lund Sweden; c Epigenetics and Diabetes Unit, Lund University Diabetes Centre, Department of Clinical Sciences, Scania University Hospital 214 28 Malmö Sweden

## Abstract

Transfecting clonal beta cells with large DNA plasmids holds significant promise for diabetes research. Existing transfection techniques like lipofection, viral transduction, and bulk electroporation often face limitations such as low efficiency and cytotoxicity. Nanoelectroporation, which utilizes nanopores or nanostraws and the application of mild electrical pulses, offers a gentle and safe alternative capable of delivering mRNAs and small to medium-sized plasmids. Nevertheless, efficiently transfecting cells with large plasmids *via* this approach remains challenging, and further improvements in efficiency are required. Here, we aimed to fill that need and optimized nanoelectroporation substrate properties to increase the transfection efficiency of GFP plasmids in clonal beta cells. We combined flow cytometry, fluorescence microscopy, and phase holographic microscopy to increase nanopore- and nanostraw transfection efficiency in terms of the delivered plasmid quantity. We found that the porosity needs to be high enough to allow the cells to interface enough nanopores, 200 nm nanopore diameter yielded higher transfection efficiency and lower cell death than 300 nm pores, and that the surface chemistry has a great effect on transfection efficiency due to differences in cell adhesion properties. Nanopores and nanostraws were compared and nanostraws were found to yield the highest immediate transfection efficiency. However, cells expressing GFP after 48 h were fewer than indicated immediately after transfection. We investigated the reasons behind this discrepancy in transfection efficiency assessed immediately- and 48 h after nanoelectroporation. Our results suggest that cells transfected with the most plasmids detach from the substrate within 48 h after transfection. This finding confirms that plasmids are cytotoxic, and it stresses the importance of achieving homogeneous transfection efficiencies among cells to be able to tune the amount of delivered plasmids appropriately.

## Abstract 2

Nanoelectroporation substrate chemistry and topography were optimized to maximize intracellular delivery of large plasmids into clonal β-cells. Delivery must be carefully tuned, as cells with high plasmid content tend to detach post-transfection.

## Graphical abstract

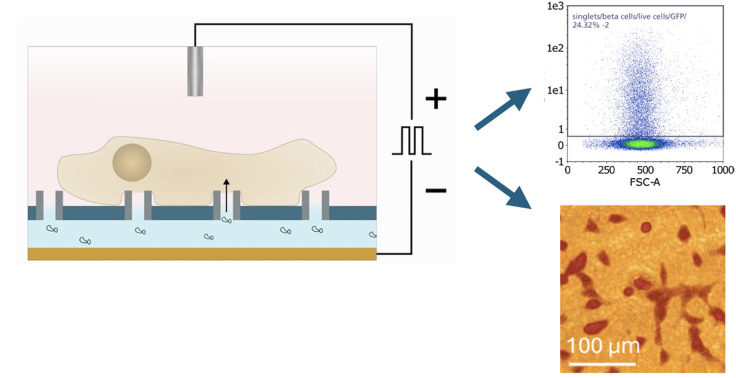

## 1. Introduction

Cell transfection remains a challenge in biomedicine as no method is free of drawbacks. Viral-mediated transfection (transduction) requires safety measures as it can elicit an immune response and insertional mutagenesis.^1,2^ Lipid-based transfection is associated with high cell toxicity^3^ and lysosomal entrapment.^4,5^ Bulk electroporation, while being efficient, leads to high cell death.^6,7^ To address these issues, new transfection methods were developed, such as nanopipettes, fluidFM or nanofountain probe electroporation.^8–11^ Although efficient, these methods are not scalable and can only address a few cells. More scalable, arrays of nanopores, nanotubes, and nanostraws have been proposed for transfecting cells, using either spontaneous membrane piercing or nanoelectroporation for opening the cell membrane and relying either on diffusion or electrophoresis for transporting the cargo inside cells.^12–21^ The advantage of using nanoelectroporation compared to bulk electroporation is that only the area of the cell membrane interfacing nanopores/straws will be electroporated, leading to a membrane recovery within minutes.^22^ We have recently suggested that nanopores could yield better transfection effects than nanostraws despite initially delivering plasmids in fewer cells and in lower amounts.^23^ Indeed, in that study, we transfected a GFP plasmid in clonal beta cells using nanostraws and nanopores of the same diameter and density and evaluated the immediate plasmid injection efficiency as well as the GFP expression 48 h later. Despite delivering more plasmids in a higher percentage of cells, nanostraw-transfection resulted in fewer cells expressing GFP after 48 h. The results could be explained by a lower cell count, which was possibly attributed to low proliferation and/or higher cell death, although the latter was not detected in flow cytometry experiments. Moreover, in the literature, various studies use substrates with different pore diameters, substrate porosities, and surface chemistries when performing nanoelectroporation.^17,24,25^ The rationale behind these choices is lacking, and no comparison of the different parameters is available. In general, there is an increasing demand for comprehensive investigation and characterization of transfection methods.^26^ In the case of nanoelectroporation, this translates to a pressing need to investigate the effects of substrate pore diameter, porosity, surface chemistry, and topography (nanopore *vs*. nanostraws) on transfection efficiency and the mechanisms behind the results. Here, we aimed to fill that need by combining flow cytometry, fluorescence microscopy, and phase holographic microscopy to optimize nanopores and nanostraws for transfection efficiency in terms of the delivered plasmid quantity. We transfected GFP plasmid in clonal beta cells and investigated substrate porosity, pore diameter, surface chemistry, and topography. Moreover, using phase holographic microscopy, we investigated the reasons behind the discrepancy in transfection efficiency assessed immediately- and 48 hours after nanoelectroporation.

## 2. Experimental

### 2.1 Substrate fabrication

**Fig. 1:**
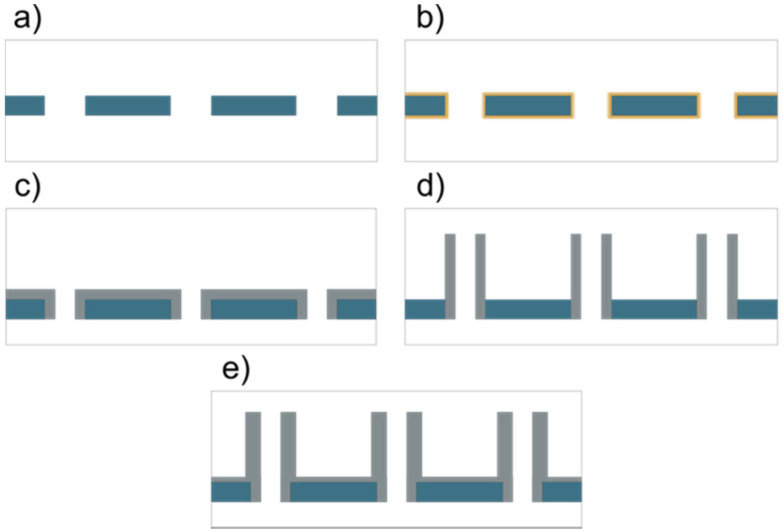
Schematics of the five substrate types that were used, with PC in dark blue, alumina in gray, and PVP in yellow. (a) As purchased PC nanopore substrate (nanopores-PC), (b) as purchased PVP-coated nanopores (nanopores-PVP), (c) alumina-coated nanopores (nanopores-Al), (d) nanostraws with polycarbonate surface between nanostraws (nanostraws-PC), and (e) nanostraws with alumina surface between the nanostraws (nanostraws-Al). The illustrations are not to scale, except for the thickness relations of alumina between the different panels.

**Fig. 2:**
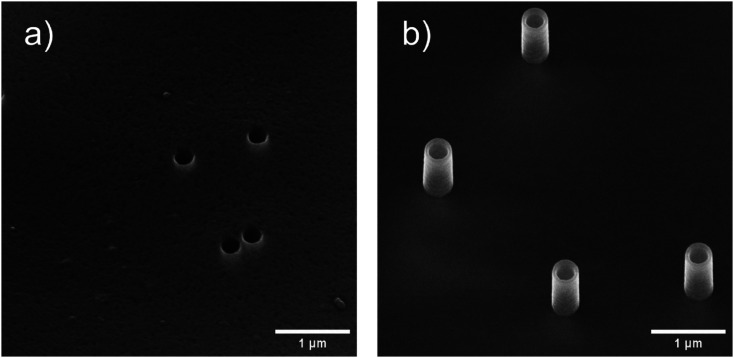
SEM images of (a) nanopores with alumina surface (nanopores-Al) and (b) nanostraws with alumina surface between the nanostraws (nanostraws-Al). The nanopores-Al have an inner diameter of ≈250 nm, and the nanostraws-Al have a height of 1 μm and inner diameter of 250 nm. In-lens detector, 30° stage tilt.

All nanopore- and nanostraw substrates were made using track-etched polycarbonate (PC) membranes coated with Polyvinylpyrrolidon (PVP), except for nanopore with PC surface chemistry. All substrates were purchased from it4ip, Belgium. They are 25 μm thick and have varying pore diameters and porosities. Five different types of substrates were made:

(a) Nanopores with PC surface chemistry: these substrates were used as purchased (hereafter called nanopores-PC, see Fig. 1a).

(b) Nanopores with PVP-coating (hereafter called nanopores-PVP, see Fig. 2b).

(c) Alumina-coated nanopores (hereafter called nanopores-Al, see Fig. 1c).

(d) Nanostraws with PC surface between the nanostraws (hereafter called nanostraws-PC, see Fig. 1d).

(e) Nanostraws with alumina surface between the nanostraws (hereafter called nanostraw-Al, see Fig. 1e).

To fabricate alumina-coated nanopores (nanopores-Al), a layer of AlO_*x*_ was deposited using atomic layer deposition (ALD, Savannah 100, Cambridge Nanotech) with alternating pulses of trimethylaluminum and water (0.15 s pulses with 30 s pump-time between) at 90 °C. To fabricate nanostraws (nanostraws-PC), the first step was to make alumina-coated nanopores, as described above, and subsequently make the substrate adhere to a 4′′ silicon wafer using an electrostatic gun (Zerostat, VWR). Thereafter, inductively coupled plasma-reactive ion etching (ICP-RIE, APEX SLR Advanced Vacuum Systems AB) was applied in two steps to expose nanostraws. In the first step, Argon (at 40 sccm, with RIE set to 60 W and ICP to 400 W) was used to remove alumina from the horizontal surfaces of the substrate, while the second step utilized O_2_ (45 sccm) and SF_6_ (5 sccm) with RIE set to 50 W and ICP to 400 W, to remove about 1 μm of PC. Both steps included helium cooling with a flow of 5 sccm. This process resulted in 1 μm high nanostraws protruding from the surface. Fig. 2 shows scanning electron microscopy (SEM) images of nanopores-Al and nanostraw-PC both with an inner diameter of ≈250 nm.

Finally, the alumina-coated nanostraw substrates (nanostraws-Al) were made by fabricating nanostraws-PC and then depositing another alumina layer using ALD. This second layer ensured the same surface chemistry over the whole substrate. However, it also increased the nanostraw wall thickness compared to nanostraws-PC substrates. The complete list of samples used in this paper and the figures where they are used can be found in Table 1.

**Table 1:** Complete list of nanopore/nanostraw substrates used in this paper

	Description	Nominal diameter	Porosity (percentage of the total area consisting of pores)	Process	Used in
1	Nanopores-PVP	200 nm	0.64%	None	Fig. 4c and 5 (top)
2	Nanopores-PC	200 nm	0.64%	None	Fig. 4c and 5 (middle)
3	Nanopores-Al	300 nm	0.06%	ALD: 130 cycles AlO_*x*_ (12 nm)	Fig. 4a
4	Nanopores-Al	300 nm	0.21%	ALD: 130 cycles AlO_*x*_ (12 nm)	Fig. 4a
5	Nanopores-Al	300 nm	0.64%	ALD: 130 cycles AlO_*x*_ (12 nm)	Fig. 4a and b
6	Nanopores-Al	200 nm	0.64%	ALD: 130 cycles AlO_*x*_ (12 nm)	Fig. 4b, c and 5 (bottom)
7	Nanopores-Al with same inner diameter as nanostraws in 8 and 9	200 nm	0.64%	ALD: 360 cycles AlO_*x*_ (33 nm)	Fig. 6–8, Fig. S7 and movie S2
8	Nanostraws-PC with same inner diameter as nanostraws and nanopores in 7 and 9	200 nm	0.64%	ALD: 260 cycles AlO_*x*_ (24 nm) then 2 step ICP-RIE	Fig. 6–8, Fig. S7 and movie S3
9	Nanostraws-Al with same inner diameter as nanostraws and nanopores in 7 and 8	200 nm	0.64%	ALD: 130 cycles AlO_*x*_ (12 nm); 2 step ICP-RIE; ALD: 130 cycles AlO_x_ (12 nm)	Fig. 6–9, Fig. S5, S7–S9 movies S4 and S5
10	Nanostraws-PC with same wall thickness as Nanostraw Al (in 9)	200 nm	0.64%	ALD: 390 cycles AlO_*x*_ (36 nm); then 2 step ICP-RIE	Fig. S5

### 2.2 SEM

After fabrication, substrates were imaged using a scanning electron microscope (SEM, LEO Gemini 1560, LEO Electron Microscopy, Inc.). Prior to imaging, a piece of the substrate was fixed on an SEM stub using carbon tape and then sputter-coated with 5 nm of Pt : Pd (80 : 20) (Q150T ES sputter coater, Quorum Technologies).

### 2.3 Cell culture

Clonal beta cells (832/13 INS-1 rat insulinoma cells), kept within a population doubling number of 24–60, were used in this study. Beta cells are insulin-producing and were chosen for their relevance to diabetes research.

The cells were cultured in RPMI-1640 medium (SH30027.01, Cytiva, HyClone) supplemented with 10% heat-inactivated fetal bovine serum (FBS, qualified, Brazil origin, Gibco), 1% penicillin–streptomycin (Sigma-Aldrich), and 2.2% supplement (50% glutamine solution, 200 mM, Gibco, 50% sodium pyruvate solution, 100 mM, Gibco, and 176 ppm 2-mercaptoethanol), referred to as culture medium. Culturing was performed in a 5% CO_2_ atmosphere at 37 °C.

When reaching approximately 80–100% confluency, the cell culture was split by rinsing with 1× Dulbecco's phosphate-buffered saline (DPBS, Thermo Fisher) before incubating with trypsin-EDTA for 3 minutes (Gibco). Trypsinization was halted by adding culture medium and followed by centrifugation at 700*g* for 3 minutes. The supernatant was removed, and the cell pellet was resuspended in fresh culture medium before a fraction of the cell suspension was seeded into a new culture flask.

### 2.4 Cargo solutions

The cargos used in this study were plasmids, pMAX GFP (3.5 kbp, prepared by the Cell & Gene Therapy Core at Lund Stem Cell Center) and eGFP (6.1 kbp, empty pcDNA3.1(+)-C-eGFP, GenScript, Netherlands), at a concentration of 0.2 mg ml^−1^ in MilliQ (MQ) water. The plasmids were stained before transfection with the intercalating dye YOYO-1 iodide (1 mM solution, Thermo Fisher) that increases 1000-fold in fluorescence intensity when bound to DNA. For staining with YOYO-1, the dye was diluted to 100 mM in MQ water and then added to the plasmid solution for a coverage of 1 YOYO-1 molecule per 250 base pairs. To ensure uniform staining, the solution was incubated at 50 °C for 2 hours.

### 2.5 Nanoelectroporation

The nanoelectroporation parameters, such as voltage, cell density and buffer conductivity were adapted to clonal beta cells and optimized in our previous study.^27^ We therefore used the parameters that were deemed to be best for transfection efficiency in the present study. For assembling the nanoelectroporation device, the nanopore or nanostraw substrate was attached to plastic cylinders (4 mm in diameter and 1 cm in height) with biocompatible double-sided tape (3M 8153LE (300LSE) double-lined Adhesive Transfer Tape), creating cell reservoirs. The tape was cut into a circular shape with a laser cutter (Epilog Laser Fusion M2) to fit on the plastic cylinder. The devices were placed in a 24-well plate and sterilized in UV light for 2 minutes before seeding the cells.

**Fig. 3:**
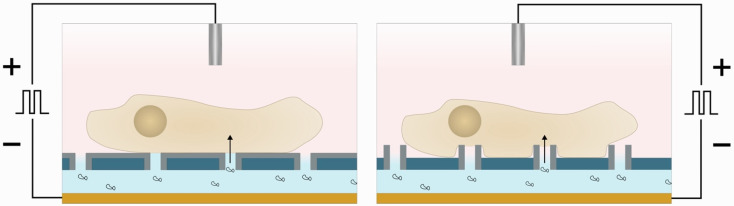
Schematic figure of a cell on a nanopore-Al substrate (left) and nanostraw-PC substrate (right). On top, the Pt electrode is inserted into the cell medium, and on the bottom is the gold electrode and cargo solution (containing plasmids). When the electric field is applied, pores are formed in the cell membrane, and the negatively charged plasmids are driven electrophoretically into the cytosol.

To seed cells in the reservoirs, the cells were resuspended using the passaging protocol described in the previous section. Using flow cytometry (MACSQuant Analyzer 16 Flow cytometer with MACSQuant running buffer, storage solution, and washing solution, Miltenyi Biotech, Bergisch Gladbach, Germany), the cell concentration was determined, and the fraction of dead cells could be evaluated by using the membrane-impermeable dye DAPI (0.01 μg ml^−1^ DAPI in MQ water, Roche, Basel, Switzerland). Culture medium was dispensed around the reservoirs in the well-plate to keep the nanosubstrate backside wet, and then 35 000 cells (2690 cells per mm^2^) were seeded in each cylinder. To ensure contact between the cells and nanosubstrate, the cylinders were centrifuged at 200*g* for 1 min. During centrifugation, all reservoirs contained 100 μl of culture medium per cell suspension. For a schematic of the cell–substrate interface, see Fig. 3.

The electroporation system consists of two electrodes – a gold-coated glass slide (100 nm Au thickness, Platypus Technologies) on the bottom and a 0.5 mm diameter platinum wire on top – connected to a pulse generator (TGP110, Aim and Thurlby Thandar Instruments, Huntingdon, UK) and an amplifier (WMA-300, Falco Systems BV, Katwijk aan Zee, Netherlands) to control the electrical pulses. An oscilloscope was used to monitor the signal.

The reservoir was dried off on the backside before being placed on top of a 15 μl drop of cargo (0.2 mg ml^−1^ plasmid in MQ water) solution on the bottom electrode. The top electrode was inserted into the cell medium in the reservoir with an inter-electrode distance of 0.5 mm. Before electroporation, 70 μl of culture medium was removed from the cylinder to minimize the contact area between the cell medium and the top electrode. The electroporation was then performed by applying a square pulse train (28 V, frequency of 40 Hz, pulse width of 200 μs) for 2 × 40 s. Between the two trains of pulses, the device was lifted and then put down again. After electroporation, the device was placed on a drop of culture medium.

To analyze the transfection results with flow cytometry, cells were resuspended by pipetting up and down in the cylinder and transferred to a 96-well plate immediately after transfection. Samples from each device were analyzed separately after being stained with DAPI (0.01 μg ml^−1^).

### 2.6 Transfection efficiency and viability

The transfection efficiency was assessed using flow cytometry, a method based on the fluorescence of individual cells. Two assays were employed: in one, the samples were analyzed immediately after transfection, made possible by the YOYO-1 dye, and in the other, the cells were cultured for 48 hours to allow for GFP expression. Cell viability was assessed using flow cytometry by adding 0.01 μg ml^−1^ of the membrane-impermeable nucleus dye DAPI to the cells before analysis.

Cells were detached from the nanosubstrate, and half of the sample was analyzed in the flow cytometer immediately, where YOYO-1 positive (*i.e.*, successfully transfected) and dead cells were recorded. Subsequently, 8000 of the remaining cells were seeded in a 48-well plate (7300 cells per cm^2^) and cultured for 48 hours. At this point, the cells were detached with trypsin (by washing cells with DPBS and adding 50 μl trypsin for 3 min) and then resuspended in 250 μl culture medium, among which 150 μl were analyzed using flow cytometry. The total sample cell count, as well as the proportion of GFP-expressing and dead cells, were estimated from the measurements. Control samples were also included, consisting of 8000 cells seeded in a 48-well plate without being in contact with the nanosubstrate before analysis.

### 2.7 Phase holographic microscopy

To study cell behavior on different types of nanosubstrates, cells were imaged using a phase holographic microscope (HoloMonitor, Phase Holographic Imaging PHI AB). The microscope and associated software (HoloMonitor App Suite) give 3D information about the cells without any perturbations since the measurements are performed in the incubator on unlabeled cells.

For imaging cells on the nanosubstrate, a piece of nanosubstrate was attached with tape at the bottom of a 35 mm Petri dish, and 850 000 cells were seeded in a total volume of 3 ml of cell medium. A special lid was used to avoid condensation (HoloLid, Phase Holographic Imaging PHI AB). The cells were left to settle at the bottom of the Petri dish for 30 minutes before the microscope settings were adjusted, and acquisition started with images taken every 10 minutes for 24 hours using the Kinetic motility assay app.

For images of cells after transfection, cells were resuspended from the cylinders after nanoelectroporation and seeded in a 24-well plate (7300 cells per cm^2^) immediately after transfection. The well plate was placed on the microscope stage in the incubator, and the cells were left to adhere for 45 minutes, after which the time-lapse imaging was initiated. During the time-lapse, images were acquired every 20 minutes for 50 hours.

### 2.8 Simulations

The electrostatic simulations of the nanostraw/nanopore membranes were done in COMSOL Multiphysics 6.3 using the electric current (EC) module. A two-dimensional model was used to lower the computational burden. To reduce the complexity of the intricate and heterogeneous cell membrane-nanostructures interaction, we modelled the cell as sitting flat on top of the nanopores/nanostraws. When a nanostraw/nanopore device is placed on the droplet of cargo solution, mixing between the cell medium (present inside the device and in the straws) and the cargo solution will occur. We assumed that the two solutions were mixed in the nanostraws and approximated the conductivity of the mixture to the average of the two solutions’ conductivity. The distance between the two electrodes was set to 500 μm, the voltage to 28 V, and the cell membrane thickness to 8 nm. The electric conductivity and relative permittivity of the different materials can be seen in Table 2.

**Table 2:** Electric conductivity and relative permittivity of the materials used in the simulation

	Cell membrane	PC membrane	Al_2_O_3_	Cell medium	0.0025× DPBS	Mix 0.0025× DPBS and cell medium	Cytoplasm
Electric conductivity (S m^−1^)	5 × 10^−7^	10^−14^	10^−14^	1.18	0.0054	0.0072	80
Relative permittivity	8	2.9	9.8	80	80	80	0.3

### 2.9 Fluorescence microscopy and image processing

Cells were stained for ki67, a proliferation marker, and imaged using fluorescence microscopy to investigate the effect of nanoelectroporation on cell proliferation. After nanoelectroporation in triplicate (see protocol above), all cells from the triplicate were resuspended and seeded together in a 24-well plate. After culturing for 24 hours, the cells were fixed by incubation with 4% paraformaldehyde for 10 minutes, followed by 3 × 5 min washing with phosphate-buffered saline (PBS). The cells were then incubated with Goat anti-Ki67 antibody solution (ThermoFisher Scientific, 1 μg ml^−1^ primary antibody in PBS with 1% BSA, 5% horse serum, and 0.25% Triton 100-x) overnight at 4 °C. Staining with anti-goat Alexa Fluor 568 secondary antibody (ThermoFisher Scientific, 4 μg ml^−1^ primary antibody in PBS with 1% BSA, 5% horse serum, and 0.25% Triton 100-x) was performed in darkness for 2 hours in room temperature. Lastly, the cells were counterstained with Hoechst 33342 (1 μg ml^−1^ in PBS) for 2 minutes. Between all staining steps, the cells were washed with PBS thrice for 5 minutes. The samples were imaged using a Nikon Ti-Eclipse microscope with an ×20 NikonFluor objective (NA = 0.45) and TRITC and DAPI filter cubes.

The images were analyzed in ImageJ software by first applying a Gaussian blur with sigma = 1 to reduce noise. The threshold was then adjusted to exclude faint cells in the case of ki67 and to include all present cells for Hoechst. The threshold for ki67 was set for all images to 7550 (full range 0–65 535). A watershed algorithm was applied to separate cells, and then the cells were counted automatically using Image J's analyzing particles feature (particle size: 4-infinity, circularity: 0.00–1.00). The images were also inspected manually to include joint cells that the watershed algorithm had missed.

For assessing GFP expression after nanoelectroporation with nanostraws-Al, the cells were treated similarly to the description in section 2.7 of phase holographic imaging of cells after transfection. The cells were resuspended after electroporation by pipetting and seeded in a 24-well plate that was then placed on the phase holographic microscope stage for 45 minutes before the image acquisition was started. After 48 hours, the well-plate was removed from the phase holographic microscope and instead imaged using a Nikon Ti-Eclipse microscope with an ×10 NikonFluor objective (NA = 0.45) and FITC filter cube. No post-processing of the images was made.

### 2.10 Statistics

All presented results include at least three independent experiments performed in triplicates. The mean of each triplicate was calculated, and the bar plots were made by averaging the mean values of a sample type from all experiments. The error bars indicate the standard error of these mean values for each sample type. A one-way ANOVA with a Tukey *post hoc* test was performed on the mean values to highlight significant differences, with *=*p* < 0.05, **=*p* < 0.01, and ***=*p* < 0.001.

## 3. Results and discussion

Various nanopore substrates were assessed in terms of transfection efficiency. The effect of membrane porosity and nominal pore diameter of the PC membranes were tested, as well as the influence of surface chemistry. In these experiments, the GFP plasmid pMAX (3.5 kbp), stained with the fluorescent dye YOYO-1, was used as cargo, and the transfection efficiency was assessed using flow cytometry.

### 3.1 Effects of substrate porosity

**Fig. 4:**
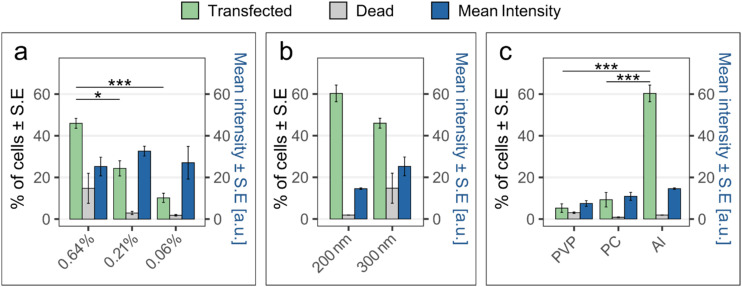
Transfection results when using nanopores of various diameters, porosities, and surface chemistries. For the corresponding flow cytometry data for transfected cells, see Fig. S1. The bars show the mean percentage ± S.E. of transfected cells (green), the percentage of dead clonal beta cells (grey), and the mean intensity ± S.E. of the transfected cells (blue) for pMAX-YOYO-1 transfection. (a) Effect of substrate porosity in alumina-coated nanopores with 300 nm nominal diameter. (b) Effect of nominal pore diameter for alumina-coated nanopores with 0.6% porosity. (c) Effect of surface chemistry using nanopore substrates with 200 nm nominal diameter and 0.6% porosity. Note that the 0.64% data in (a) and the 300 nm data in (b) are the same data, as well as the 200 nm data in (b) and the Al data in (c). (*n* = 3, the statistics were calculated with ANOVA and Tukey Post Hoc test: ***p < 0.001, *p < 0.05).

The effect of substrate porosity was tested on PC membranes of 300 nm nominal pore diameter coated with 12 nm alumina. Three porosities were evaluated: 0.64%, 0.21%, and 0.06% (Fig. 4a). The results show that the higher the porosity, the higher the percentage of transfected cells. That can be explained by the average distance between nanopores for the different densities, *i.e.*, 3.3 μm for the membranes with 0.64% porosity, 5.8 μm for the membranes with 0.21% porosity, and 11.2 μm for the 0.06% porosity membranes. Considering that the pores are distributed randomly on the substrate and the quite small size of the INS-1 cell size (≈10 μm), it is possible that on the low porosity substrates, only a few cells are interfaced with enough nanopores to be transfected with a detectable amount of plasmid. On the other hand, cells successfully transfected with the two lower substrate porosities have a higher fluorescence intensity, suggesting that more plasmids were delivered in these cells. This can be explained by the higher electric field across each nanopore resulting from the low substrate porosity.

### 3.2 Effects of nanopore diameter

By comparing alumina-coated PC membranes with 0.6% porosity but different nominal pore diameters (200 nm and 300 nm), the dependence of transfection efficiency on nominal pore diameter could be determined. For the smaller diameter, the results show a higher percentage of transfected cells and a lower percentage of dead cells than when using 300 nm nominal diameter nanopores (Fig. 4b). The higher cell death observed when using 300 nm nanopores is possibly due to a more difficult cell recovery when larger pores are formed in the cell membrane. However, using a smaller diameter also resulted in fewer plasmids delivered in the cells, as shown by their lower fluorescence intensity. The higher intensity and proportion of dead cells for the 300 nm nominal diameter nanopores is possibly due to a greater surface area of the cell membrane being electroporated, resulting in more plasmids being delivered, but also to an uncertain recovery of the cell membrane integrity.

### 3.3 Effect of surface chemistry

Next, the effect of surface chemistry was investigated by using nanopore substrates with the porosity and nominal pore diameter yielding the best results so far, *i.e.*, 0.6% porosity and 200 nm nominal diameter. Three types of surface chemistries were tested: bare PC, alumina (used in 3.2 above), and PVP. These results show that compared to alumina-coated membranes, very low transfection efficiency is achieved with both PC surface and PVP-coated membranes (Fig. 4c).

To achieve good transfection efficiency, a tight seal between the cell membrane and the nanopore should be formed, followed by the application of an electric field strong enough to destabilize the cell membrane and drive the plasmids to the cytosol.

Previous studies have reported coating PC nanopore membranes with adhesion-promoting molecules, such as fibronectin, before electroporation,^16,17,24^ suggesting cell adhesion is a key factor in achieving efficient transfection. In these studies, the cells were cultured for hours on the nanopore substrate before transfection, in contrast to here, where the cells were gently spun down and transfected within minutes after that. Despite the short time between spinning down cells and transfecting them, cells interact and start adhering to the substrates immediately after being spun down. Therefore, it is possible that transfection efficiency is affected by the early stages of cell adhesion on the substrate. Hence, we investigated a potential correlation between the poor transfection efficiency of bare PC and PVP-coated nanopore membranes and the cell adhesion on these substrates.

**Fig. 5:**
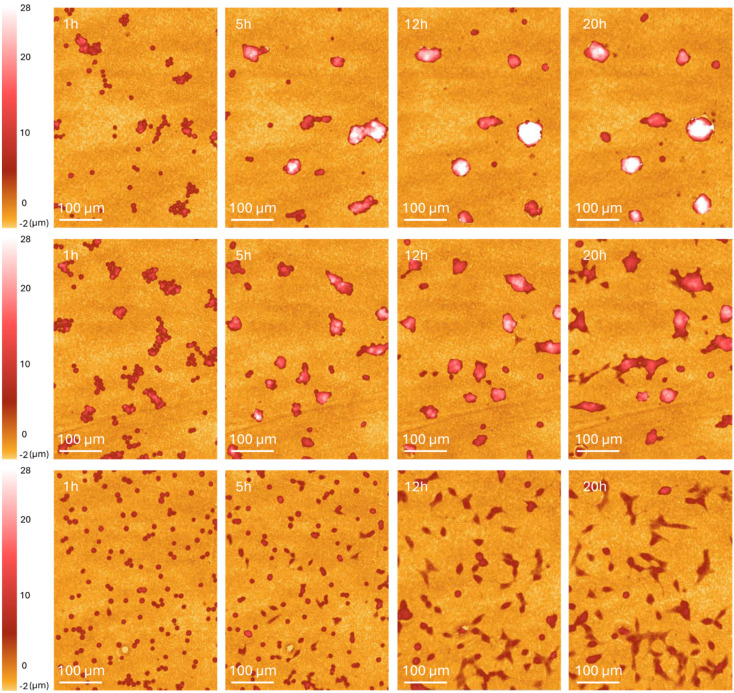
Time-lapse images of clonal beta cells cultured on track-etched membranes using phase holographic microscopy inside the cell incubator. Top: nanopore-PVP, middle: nanopore-PC bottom: nanopore-Al. The starting point of all three substrates were 0.628% porosity membranes with 200 nm pore diameter.

To investigate cell adhesion, the cells were seeded on PVP-coated nanopore membranes, PC nanopore membranes, and alumina-coated nanopore membranes, all with a nominal pore diameter of 200 nm. Time-lapse images were taken every 10 min for 20 hours using Phase holographic microscopy. When cultured on PC- and PVP-coated nanopore membranes, cells form aggregates that grow perpendicularly to the substrate already after 5 hours (Fig. 5a and b). This shows a poor cell affinity for the substrate. In contrast, cells cultured on alumina-coated nanopores spread individually on the substrate without any tendency to aggregate (Fig. 5c), similar to when cultured on standard culture substrates (see Fig. S3). The results suggest that using PC- or PVP-coated substrates results in poor cell adhesion, as cells minimize contact with the substrate. One possible explanation for the higher transfection efficiency obtained using nanopores-Al could be the higher field strength resulting from a smaller diameter than PC and PVP-coated nanopores (∼24 nm smaller due to the alumina coating). However, this can be dismissed since nanopores-Al with a nominal pore diameter of 300 nm (resulting in a much larger diameter than the PVP and PC nanopores) yield better results than PVP and PC nanopores (Fig. 4a). Hence, transfection efficiency seems to depend on surface chemistry, where PVP and PC lead to poor transfection. For comparison, we transfected cells with a PC nanopore substrate coated with fibronectin. Surprisingly, the transfection efficiency was low, at a level similar to the one achieved with PC and PVP substrates (Fig. S2). This could be due to possible differences in cell adhesion kinetics on the different substrates.

### 3.4 Improved initial transfection efficiency using nanostraws

The results indicate that coating the PC substrate with alumina dramatically improves transfection efficiency. Another efficient transfection tool based on PC track-etched membranes is nanostraws, *i.e.*, alumina nanotubes protruding from the PC membrane. Nanostraws are fabricated by etching the top horizontal alumina layer from a nanopore-Al substrate, such as the ones described above, and subsequently etching the PC to obtain protruding nanostraws (nanostraws-PC). Considering the positive effect of alumina coating on transfection efficiency reported above, one can wonder whether coating the PC surface between the nanostraws with alumina (*i.e.*, making nanostraws-Al) would improve the transfection efficiency further.

**Fig. 6:**
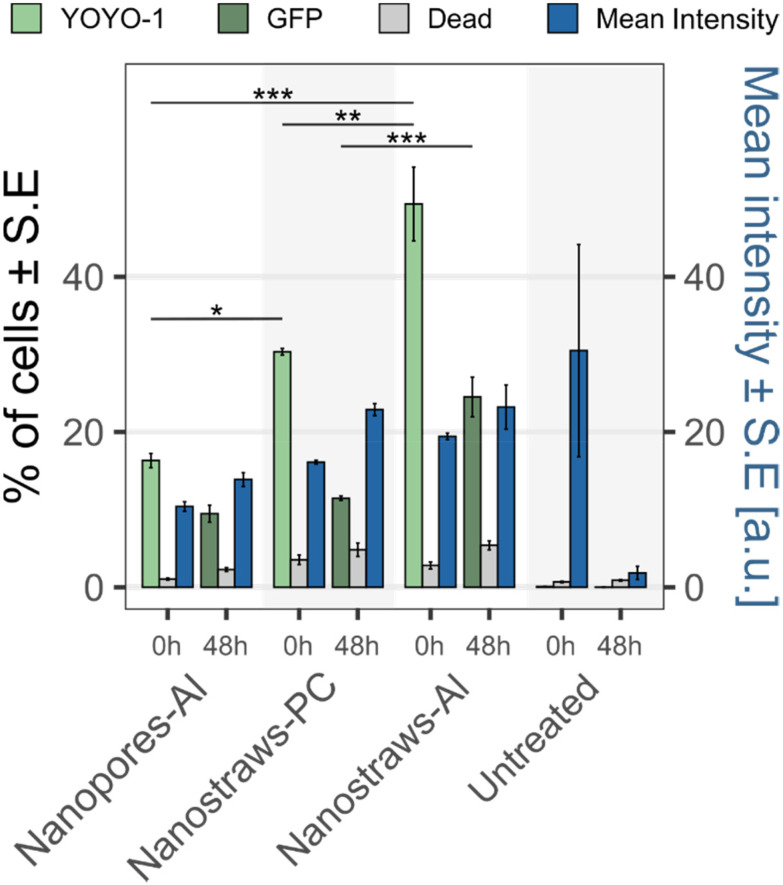
Transfection of eGFP plasmid stained with YOYO-1 using nanopores-Al, nanostraws-PC, and nanostraws-Al (all with similar inner diameter). A part of the clonal beta cells in each sample was analyzed for YOYO-1 fluorescence immediately after transfection, and 8000 cells were seeded and cultured for 48 hours before being resuspended and analyzed for GFP expression. The bars show the mean percentage ± S.E. of transfected cells (green), percentage ± S.E of dead cells (gray), and the mean intensity ± S.E. of the transfected cells (blue) for eGFP-YOYO-1 transfection at 0 h and the corresponding GFP expression after 48 h. Due to different fluorescence intensity levels for EGFP-YOYO-1 and GFP, the flow cytometry gain is not the same for the two fluorochromes, and the intensity at the two time points should not be compared. For the corresponding flow cytometry data for YOYO-1 and GFP cells, see Fig. S4. The seemingly high intensity at 0 h of the untreated sample is due to the fact that the mean intensity is calculated solely from positive cells, and in these samples, only 1–5 cells were fluorescent (except for one sample, where 20 cells were fluorescent). (*n* = 3, the statistics were calculated with ANOVA and Tukey Post Hoc test: ***p < 0.001, **p < 0.01, *p < 0.05. The complete list of statistical significances can be found in Table S1).

To investigate this, nanopores-Al, nanostraws-PC, and nanostraws-Al were fabricated from the same type of track-etched membrane (200 nm nominal pore diameter and 0.64% porosity), with a similar final inner diameter of 122 ± 12 nm and a height of 1 μm for the nanostraws. Note that, due to fabrication constraints, the wall thickness of the nanostraws-Al is thicker compared to the walls of nanostraws-PC (with a deposition of 36 nm alumina compared to 24 nm). The eGFP plasmid (6.1 kbp), coding for green fluorescent protein (GFP), was chosen as cargo because of the need for larger cargo in future applications. The plasmid was stained with YOYO-1 and injected into cells using the three substrates described above. The transfection outcome was evaluated immediately after transfection by assessing the YOYO-1 fluorescence, as well as after 48 h by assessing the expressed GFP fluorescence in the cells (Fig. 6). Note that the fluorescence intensity of GFP is much higher than that of YOYO-1, such that a lower gain is used in flow cytometry when probing cells for GFP expression. Therefore, hypothetical YOYO-1-positive cells would be excluded from the GFP-positive cell count by the gating process in flow cytometry.

When assessed immediately after transfection, the percentage of transfected cells and their mean intensity are higher for transfection with nanostraws compared to alumina-coated nanopores of similar diameter (Fig. 6). Interestingly, the fraction of transfected cells increased further when the nanostraw substrate was fully coated with alumina (nanostraws-Al). When instead looking at the percentage of GFP-expressing cells after 48 h, the same trend can be seen, although, for each substrate, a smaller percentage of cells was positive for GFP compared to the initial values immediately after transfection. Note that the mean intensity of the untreated sample immediately after transfection is high. However, the mean intensity is calculated solely from positive cells, and since there are only 1–5 (20 cells in one case) positive cells in these samples, the average is calculated from outliers.

The higher transfection efficiency obtained when using alumina-coated nanostraws may be due to either the alumina surface between the nanostraws or the increased nanostraw wall thickness. To investigate this, nanostraws were made with the same wall thickness as the alumina-coated nanostraws but with PC between the nanostraws. For this substrate, the transfection efficiency immediately after nanoelectroporation was lower than for the alumina-coated nanostraws (Fig. S5), which suggests that having alumina between the nanostraws is vital.

Another factor behind the observed differences between the three substrates could be a possible difference in the electrical potential across the substrate, which would impact the electrophoretic force driving the plasmids to the cytosol. Simulations of the electric field distribution across the different substrates show that the electric potential is very similar (Fig. S6), with the voltage dropping inside the pore from top to bottom. Thus, the observed difference in transfection immediately after injection is not due to a difference in electric potential between the substrates.

### 3.5 Investigation of the lower-than-expected transfection efficiency when assessed 48 h after transfection

**Fig. 7:**
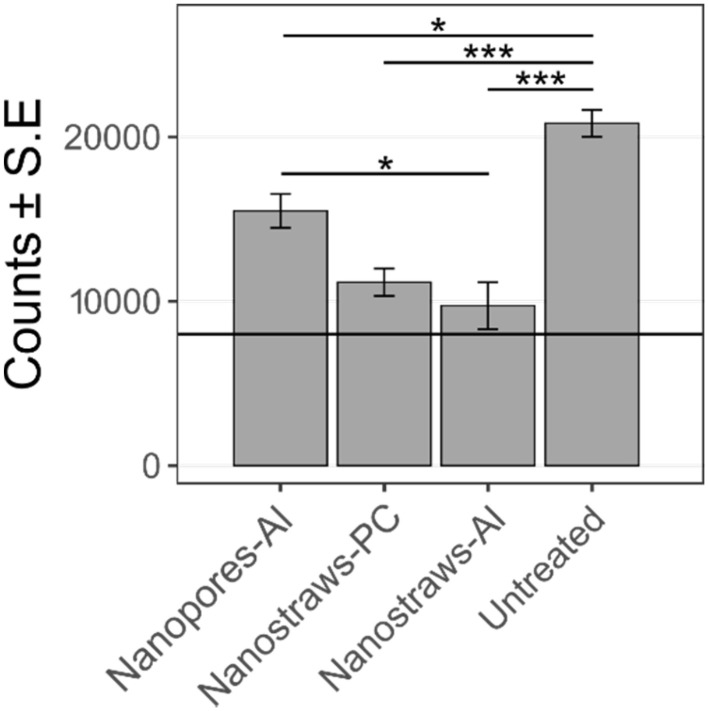
Cell counts measured 48 h after transfection using the various nanosubstrates. Immediately after transfection, 8000 clonal beta cells (horizontal black line) were seeded in a 48-well plate and incubated for 48 h before being counted using flow cytometry (*n* = 3, the statistics were calculated with ANOVA and Tukey Post Hoc test: ***p < 0.001, *p < 0.05).

For all investigated substrates, especially nanostraws-Al, a lower percentage of cells was found to express GFP after 48 h compared to the initial proportion of YOYO-1 positive cells (see Fig. 6). This can have multiple explanations. The first one would be that cells divided, and the plasmids were “diluted” between daughter cells, resulting in the fluorescence of some cells being below the detection threshold. The second would be that cells divide less after nanoelectroporation. Indeed, since plasmids need to translocate to the nucleus for GPF to be expressed, which mainly occurs during the telophase at the end of cell division,^28^ reduced cell proliferation would result in fewer cells expressing GFP. The third explanation would be significant cell death, although this was detected neither immediately (low amount of cell death in Fig. 6 and a similar cell count as the control immediately after transfection, see Fig. S7) nor 48 hours after nanoelectroporation. To investigate this, a cell count was performed 48 hours after transfection with the various substrates. Immediately after transfection, 8000 live cells were seeded in a 48-well plate, represented by the black horizontal line in Fig. 7. After 48 h, in contrast to all transfected samples, where lower cell counts were observed, the untreated cell population had more than doubled. The lowest cell count was seen for nanostraws-Al, followed by nanostraws-PC and nanopores-Al.

**Fig. 8:**
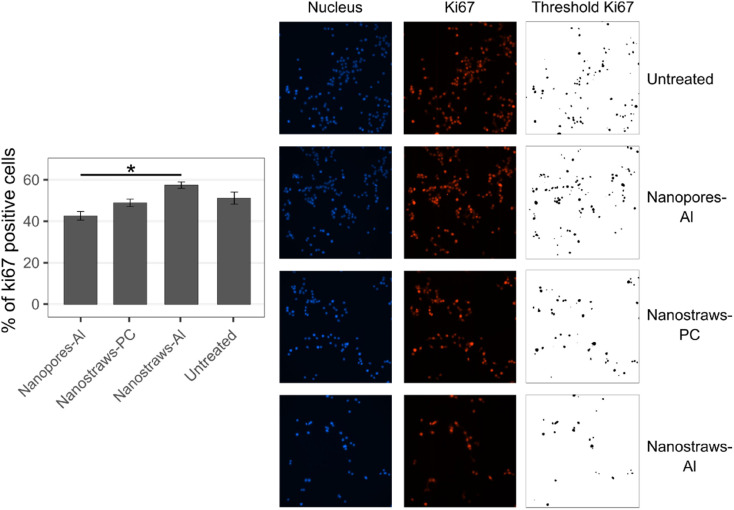
ki67 immunostaining of clonal beta cells 24 h after nanoelectroporation with the three substrates. The same threshold was set for all images of ki67, the error bars indicate ± standard error, and ANOVA was done with the mean values from the three experiments, *: *p* < 0.05.

The lower cell count compared to controls can be attributed to cell death and/or lower cell proliferation. To investigate the effect of cell proliferation, we assessed cells for the presence of the proliferation marker ki67 24 h after nanoelectroporation, see Fig. 8.

Surprisingly, the cells transfected with nanostraws-Al had the highest proportion of proliferating cells despite the cell count for these cells being the lowest after 48 h. This would suggest that cell death is high, which is not what was measured using flow cytometry (neither immediately nor 48 h after nanoelectroporation. See Fig. 6). A possible explanation is that, when dying, cells quickly detach from the substrate and are rinsed off when preparing the sample for flow cytometry. Alternatively, the cells adhere poorly to the substrate.

**Fig. 9:**
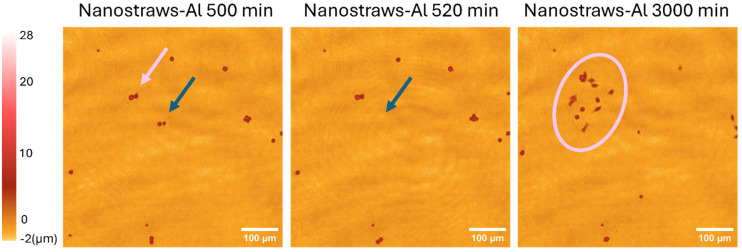
Representative phase holographic images showing clonal beta cells transfected using nanostraws-Al and subsequently seeded in 24-well plates. The images show the cells 500 min, 520 min, and 3000 min after starting the time-lapse imaging. The blue arrows show the detachment of 2 cells from the substrate shortly after cell division. The pink arrow shows two cells that were able to attach, spread, and divide on the substrate, giving rise to a small group of cells, highlighted in pink in the 3000 min image. See movie S4 for the complete sequence of events.

To investigate this, cells were transfected using the various substrates and seeded in a 24-well plate for 45 min before time-lapse imaging was initiated using phase holographic microscopy. Images were taken every 20 min for 50 h. Note that in these experiments, the seeding cell density was chosen to be the same as in the cell count experiments (Fig. 7) to avoid any possible influence of cell confluence on the results at 48 h.^27^ Therefore, the number of cells visible in each image is low and cannot be used for quantitative analysis. In all samples, some cells can be seen “floating away” above the substrates (see movies S1–S4). The movement of the non-attached cells is due to the stage movement of the microscope when acquiring images of the different samples. All cells are subjected to the same movement since they are placed in the same well plate. Noticeably, cells that were transfected using nanostraws-Al and are close to immobile disappear from the field of view between 2 consecutive images, suggesting a detachment (see movie S4 and Fig. 9). The detachment often happens after cell division, suggesting that these cells are not forming enough- or strong enough bonds with the substrate to ensure proper respreading on the substrate after mitosis. A small subset of cells is able to attach, spread, and divide on the substrate, forming a small group of cells (pink circle in Fig. 9). This shows that there are two cell populations, with most cells unable to adhere and a small number of cells behaving normally on the substrate. Since we observed this for nanostraws-Al, which gives the best initial transfection efficiency, we can hypothesize that cells successfully transfected with nanostraws-Al are detaching from the substrate. This theory is supported when assessing the fluorescence of cells 48 h after being transfected with nanostraws-Al, where the cells expressing most GFP are rarely part of the small clusters of cells having divided successfully, such as the one circled in Fig. 9 (see Fig. S8). On a side note, this implies that, when assessing the cell proliferation after 24 h (Fig. 8), most assessed cells belonged to the group of cells with little GFP expression and were able to attach and divide. Why these cells tend to proliferate more than untreated cells is unclear.

In contrast to cells transfected using nanostraws-Al, untreated cells can be seen dividing with daughter cells remaining in the vicinity of the location where the division took place. This results in small cell clusters on the substrate after 48 h (see Fig. S9 and movie S6 for the complete sequence of events). Moreover, cells transfected by “mock” nanoelectroporation with nanostraws-Al (same nanoelectroporation protocol but no plasmid) show similar behavior to untreated cells, with cell spreading on the substrate and no cells detaching from it (see Fig. S9 and movie S5 for complete sequence of events). Combined, these results suggest that the internalized plasmids are responsible for the cell detachment from the substrates, not the nanoelectroporation process *per se*. This agrees with a previous study investigating the toxicity of plasmids as a function of size when delivered using bulk electroporation.^29^ That study reported cell detachment from the substrate after electroporation, to an extent scaling with the plasmid size and in a way that is independent of the amount of plasmid delivered in the cells. In our case, the eGFP plasmid size is ≈6 kbp, and we have previously reported a lower cell count for cells transfected with the pMAX plasmid (≈3.5 kbp) using nanopores-Al and nanostraws-PC compared to controls (untreated cells).^27^ Therefore, our results suggest that the toxicity is not due to plasmid size, at least in the range of 3–6 kbp. On the other hand, in our case, substrates showing the best transfection efficiencies (nanostraws-Al and nanostraws-PC) led to the lowest cell counts after 48 h due to poor cell adhesion. We can, therefore, speculate that it is the amount of transfected plasmid which governs the observed toxicity.

The toxicity could be addressed by lowering the plasmid solution concentration or reducing the voltage driving the plasmids to the cytosol. That is, however, not a solution with the substrates used here since not all cells are transfected, and there is a huge variation in the quantity of plasmids delivered between cells. Ways to address the uneven plasmid distribution among cells are not straightforward, especially when using nanosubstrates made from track-etched membranes, where the pores are distributed randomly on the substrate. Using substrates with nanotubes, nanoneedles, or nanopores arranged in arrays could potentially improve the transfection homogeneity between cells.^30–32^

## 4. Conclusions

Clonal beta cells have been transfected with GFP plasmids by nanoelectroporation using different nanopore- and nanostraw substrates. Transfection efficiency was assessed immediately after transfection by evaluating the amount of plasmids transported to the cells and 48 hours after transfection by analyzing the GFP expression in the cells. The results show that cells could not be transfected using “as purchased” nanopores (made of PC, with or without PVP coating) due to poor cell adhesion. However, coating the substrate with alumina dramatically improved the transfection efficiency. For similar substrate porosities, using a larger pore diameter (300 nm instead of 200 nm) led to increased cell death after transfection, possibly due to challenging cell recovery when larger pores are formed in the cell membrane with nanoelectroporation. Using nanostraws-PC (alumina nanostraws with PC surface chemistry between the nanostraws) and particularly nanostraws-Al (nanostraw substrates entirely covered with alumina) resulted in higher transfection efficiency than nanopores. However, the number of cells 48 h after transfection was significantly lower for all transfected substrates compared to untreated cells, an effect most pronounced for cells transfected with nanostraws-Al. After investigation using phase holographic microscopy, cells transfected using nanostraws-Al could be seen detaching from the substrate, and only a few cells attached to the substrate and proliferated. This stands in contrast to untreated cells and cells undergoing nanoelectroporation devoid of plasmid. This poor cell adhesion shows that detachment of cells is the most probable explanation for the lower cell count. Moreover, it explains why the missing cells are not included in the flow cytometry dead cell count, as the cells are washed once before being collected. Using fluorescence microscopy, we were able to correlate good cell adhesion after transfection using nanostraws-Al with low or no GFP expression, which suggests that the observed toxicity is not due to nanostraw electroporation but to the plasmids themselves. Therefore, the amount of plasmid delivered in cells should be tuned to avoid cytotoxicity. However, that is difficult to achieve with randomly distributed nanopores and nanostraws, as different numbers of nanopores and nanostraws interface each cell, and where some cells are not transfected while others are injected with toxic levels of plasmids. Possible solutions to this could be to use substrates with nanopores or nanostraws distributed evenly in an array, so that each cell interfaces the same number of nanopores/nanostraws, and to lower the plasmid concentration in the cargo solution. In addition, our results stress the importance of considering the cell count in addition to focusing on the percentage of live cells transfected when using flow cytometry.

## Conflicts of interest

There are no conflicts to declare.

## Supplementary Material

NR-017-D5NR02352A-s001

NR-017-D5NR02352A-s002

NR-017-D5NR02352A-s003

NR-017-D5NR02352A-s004

NR-017-D5NR02352A-s005

NR-017-D5NR02352A-s006

## Data Availability

Data is available on the Zenodo repository: https://zenodo.org/records/15577149?token=eyJhbGciOiJIUzUxMiJ9.eyJpZCI6IjkyMDNjYTc5LTZkNzEtNDZkNy05MTllLWNkNGY5OTRjMjRhNyIsImRhdGEiOnt9LCJyYW5kb20iOiI1YTZhYTVhNDJlMTkyZDIyOGIyMDA4ODdlOGNlYzc5ZSJ9.JWy1OqudpdU_MJRqu2-2OmkCGWHLs6pHW9DC5yZq5BrrWrdCcWxIxp9FIi6djsGBDTuCdAwhdkptfJtDWO7n-w. Supplementary information is available: Flow cytometry raw data, transfection results on additional controls, and Phase holographic imaging movies. See DOI: https://doi.org/10.1039/d5nr02352a.
