# Supplementary material for: Plasmid-induced cytotoxicity revealed by nanopore and nanostraw electroporation
Source: Nanoscale. 2025 Sep 8;17(38):22382–93. doi: 10.1039/d5nr02352a (PMC12434679; doi:10.1039/d5nr02352a)
Supplement: NR-017-D5NR02352A-s006 [file NR-017-D5NR02352A-s006.pdf]

This journal is © The Royal Society of Chemistry 2025

Plasmid-induced cytotoxicity revealed by nanopore and nanostraw electroporation.

Frida Ekstrand [a,b], Sara Davidsson Bencker [a,b], Sabrina Ruhrmann [c], Yupeng Yang [a,b], Charlotte Ling [c], Christelle N. Prinz [a,b] *

[a] Division of Solid State Physics, Lund University, 221 00 Lund, Sweden

[b] NanoLund, Lund University, 221 00 Lund, Sweden

[c] Epigenetics and Diabetes Unit, Lund University Diabetes Centre, Department of Clinical Sciences, Scania University Hospital, 214 28, Malmö, Sweden

* Corresponding author: christelle.prinz@ftf.lth.se

SUPPLEMENTARY INFORMATION

Figure S1

Flow cytometry data (density plot) for the cells shown in the green bars in Figure 4. All cells subjected to the same conditions were gathered into one plot. FSC-A: Forward scattering, area of the signal; YOYO-A: YOYO-1 fluorescence in cells, area of the signal.

a) Porosity

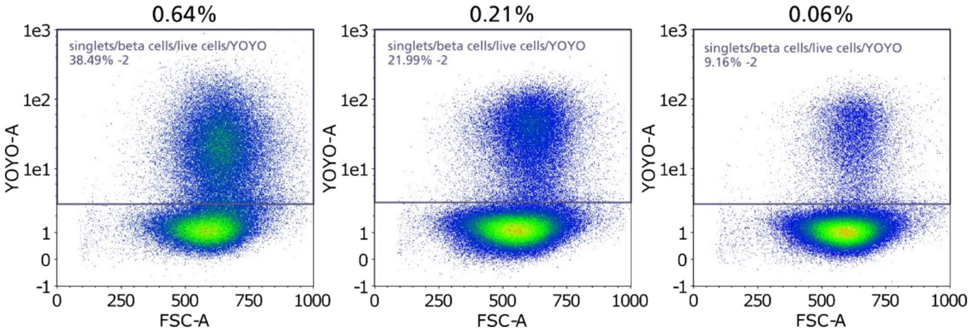

b) Nanopore diameter

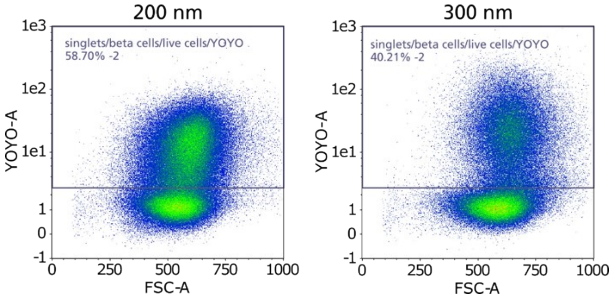

c) Surface chemistry

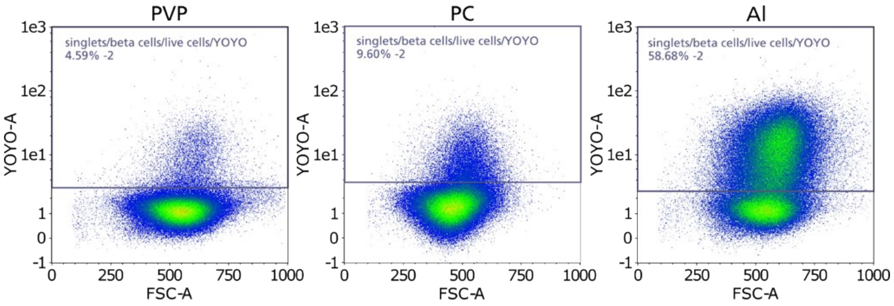

Figure S2

Transfection efficiency assessed immediately after nanoelectroporation using a fibronectin-coated PC membrane with 200 nm pore diameter and 0.64% porosity.

For fibronectin coating, the devices were filled with 20 ul of 100 ng/ml human fibronectin (Corning, Fisher Scientific) (stock solution of 1 mg/ml was diluted in MQ water), before incubation at 37 °C for 4 hours. The devices were subsequently rinsed twice with MQ water and once with cell medium.

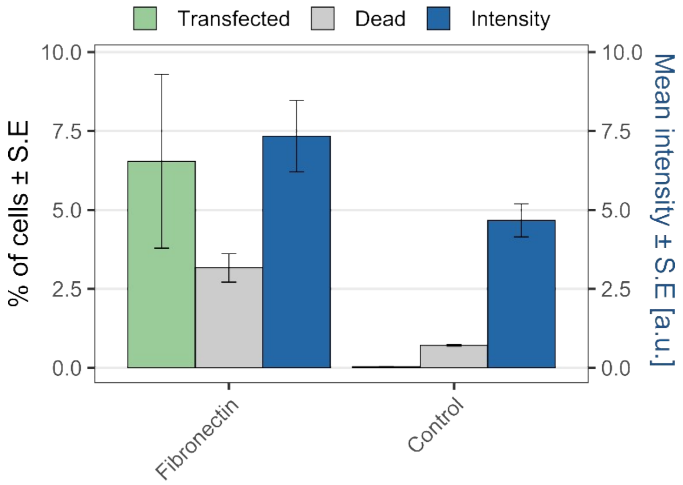

Figure S3

Phase holographic time lapse images of clonal beta cells cultured on standard culture substrates.

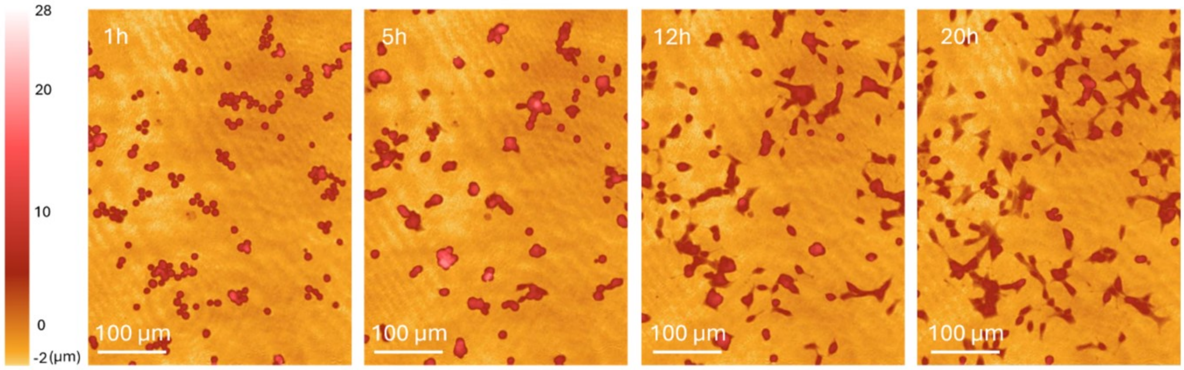

Table S1

Significance levels between the different substrates in Figure 6 for the parameters investigated (YOYO-1, GFP, Mean intensity). The statistics were calculated with ANOVA and Tukey Post Hoc test on the mean values from each experiment: ***$p < 0.001$, **$p<0.01$, *$p < 0.05$.

Substrates	Parameter	Significance
Nanostraws-Al and nanopores-Al	YOYO-1	***
Nanostraws-PC and nanopores Al	YOYO-1	*
Nanostraws-Al and nanostraws-PC	YOYO-1	**
Nanostraws-Al and nanopores-Al	GFP	***
Nanostraws-Al and nanostraws-PC	GFP	***
Nanostraws-Al and nanopores-Al	Mean intensity 0h	*

Figure S4

The flow cytometry data for the plasmid transfected cells in Figure 6, all individual samples for one condition combined into the same plot.

a) YOYO-1

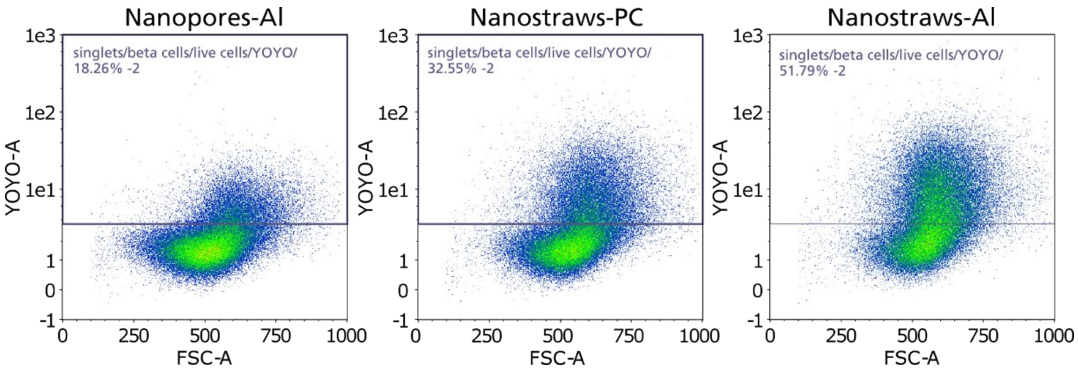

b) GFP

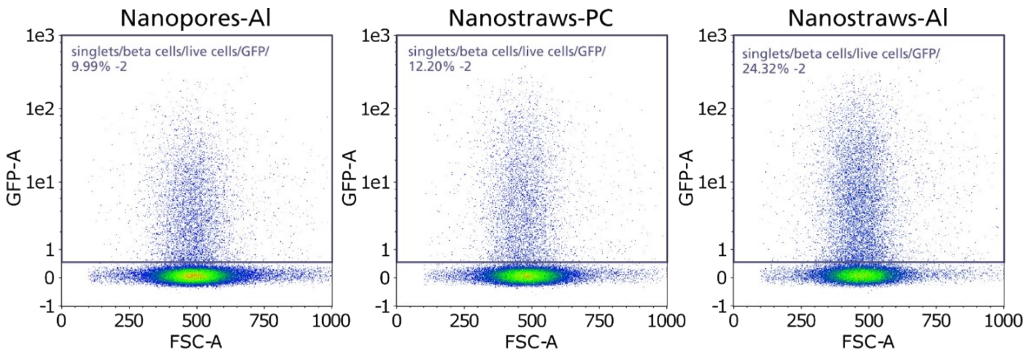

Figure S5

Transfection efficiency assessed immediately after nanoelectroporation for nanostraws with the same nanostraw wall thickness but different surface chemistries between the nanostraws. PC: Nanostraws-PC and Al: Nanostraws-Al (also shown in Figure 6). Due to redeposition during PC etching process, both nanostraw types have around 45 nm wall thickness. T-test, *p=0.0108 (done on all individual data points)

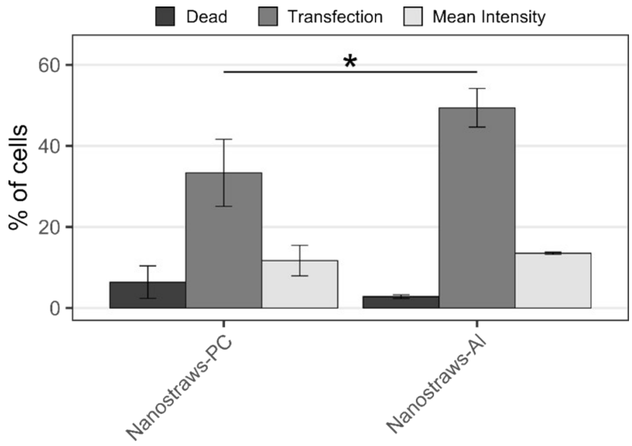

Figure S6

Simulations of the electric potential (Volts) during nanoelectroporation across the alumina-coated nanopore-, nanostraw- and alumina-coated nanostraw substrates, with a cell on top, when pores have been opened in the cell membrane.

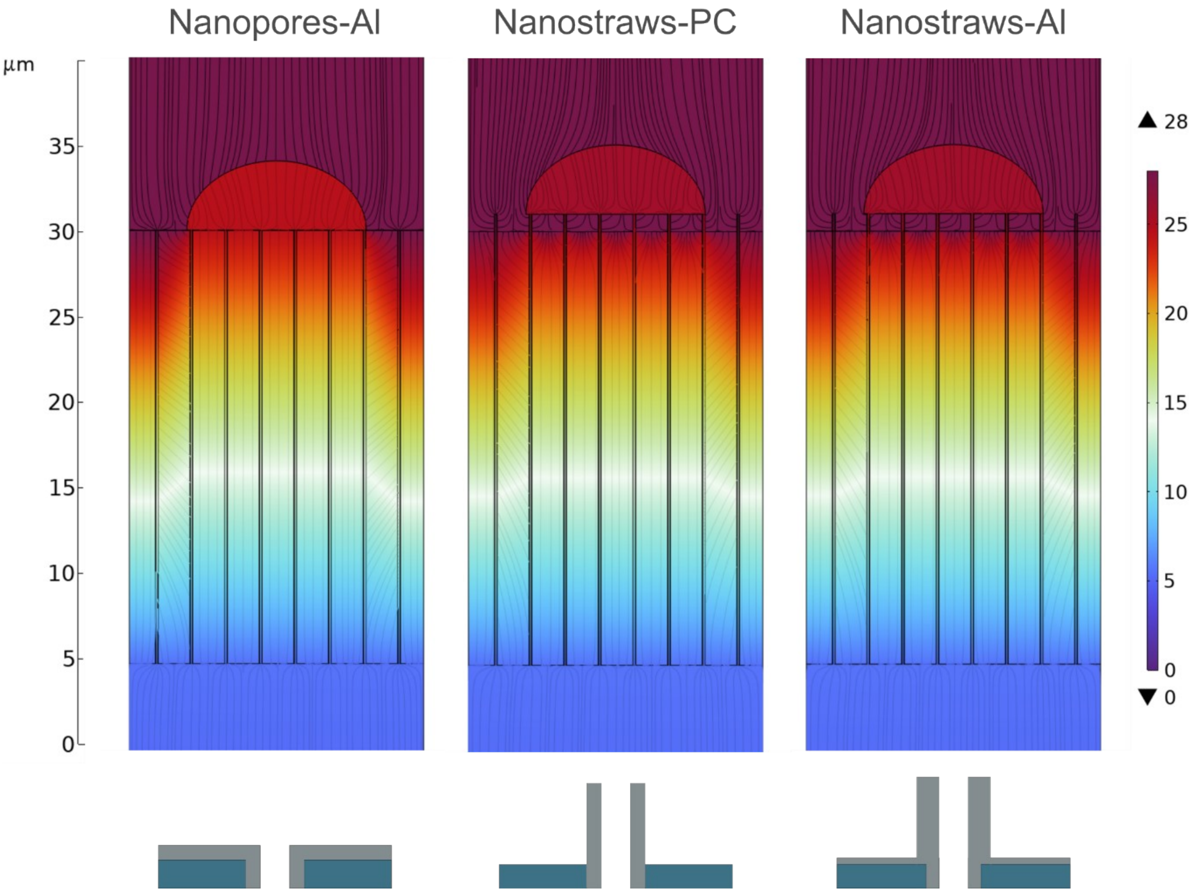

Figure S7

Cell counts immediately after nanoelectroporation for the substrates investigated in Figure 6. The total sample volume was 240 µl, of which 120 µl were taken for flow cytometry analysis, of which 80 µl were actually measured. The cell number measured in the 80 µl was then multiplied by 3 to obtain the cell count of the whole sample (35 000 cells were seeded in the devices before nanoelectroporation)

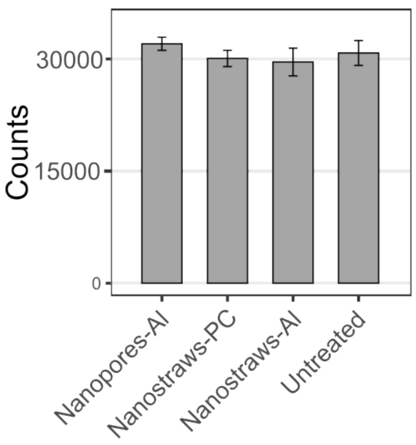

Counts
30000
15000
0
Nanopores-Al
Nanostraws-PC
Nanostraws-Al
Untreated

Figure S8

GFP expression of cells transfected using nanostraws-Al, assessed using fluorescence microscopy 48 h after transfection. Representative bright field images (left) and corresponding fluorescence (right). The majority of cells that have divided and attached form small clusters of cells and have low or no GFP fluorescence.

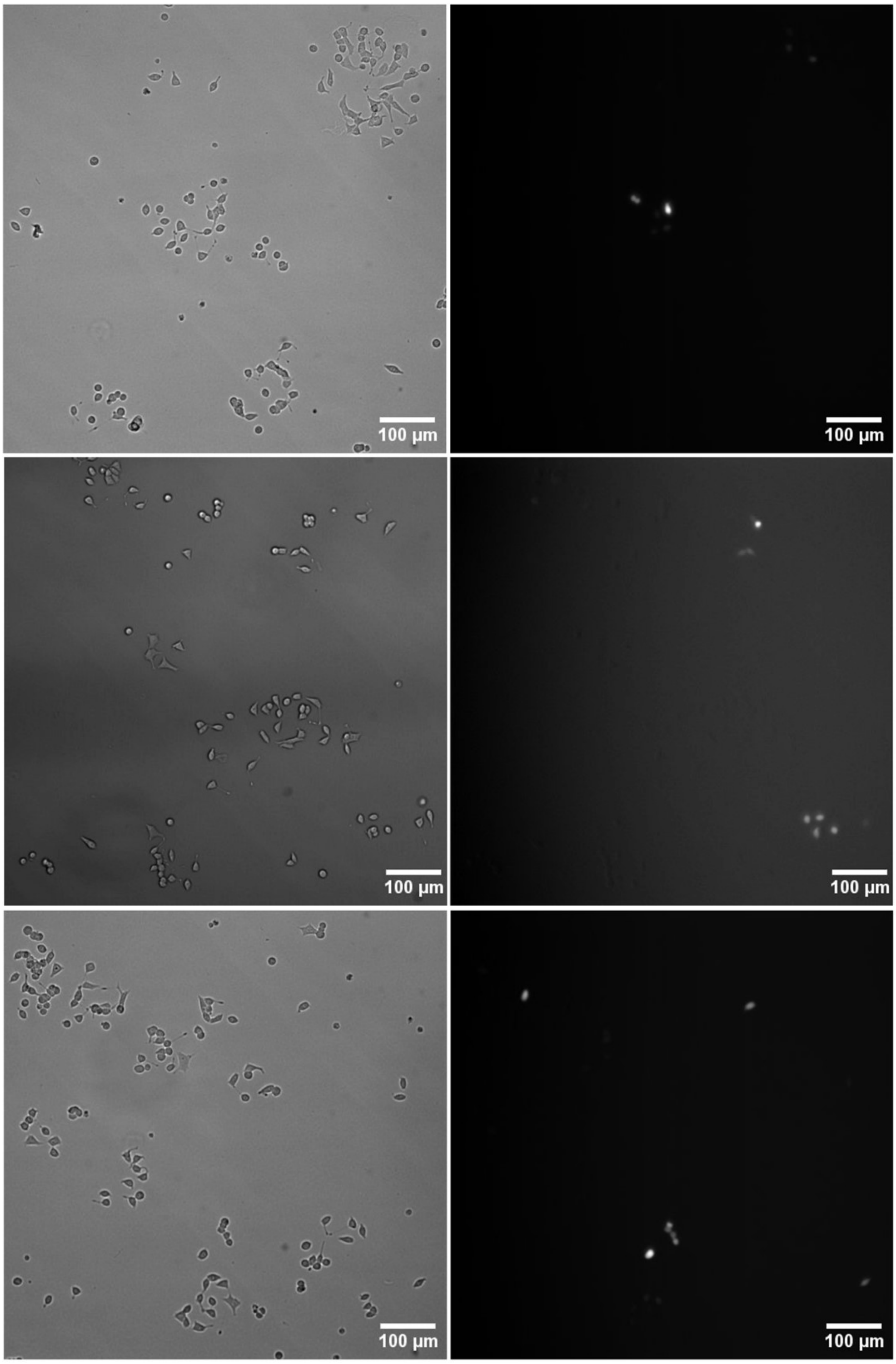

Figure S9

Representative phase holographic images of untreated clonal beta cells and cells undergone mock nanoelectroporation nanostraws-Al (same protocol but no plasmids), seeded in 24-well plates, 48 h after starting the time-lapse imaging.

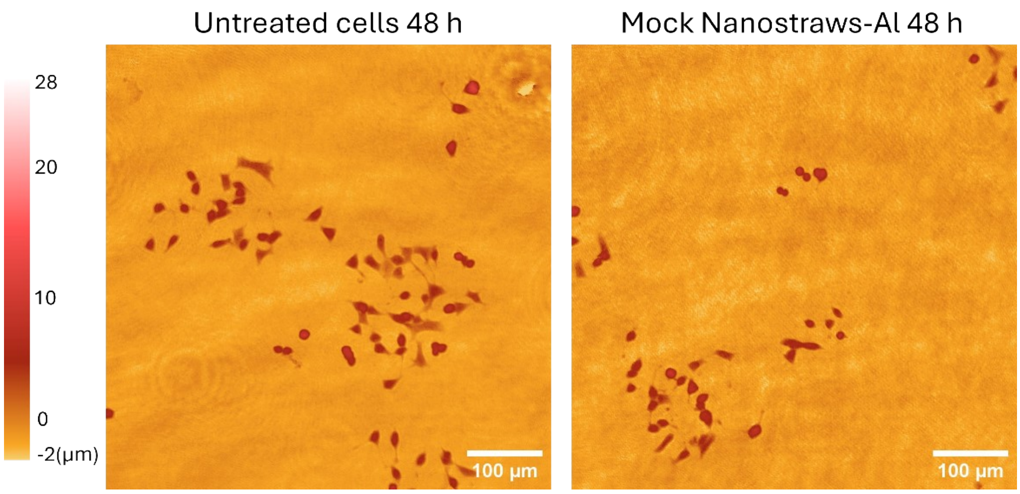

Movie S1

Phase holographic movie of untreated cells seeded in a 24-well plate at a concentration of 7300 cells/cm^2. Images were acquired every 20 minutes for 50 hours.

Movie S2

Phase holographic movie of cells transfected using nanopores-Al, seeded in a 24-well plate after nanoelectroporation (7300 cells/cm^2). Images were acquired every 20 minutes for 50 hours.

Movie S3

Phase holographic movie of cells transfected using nanostraws-PC, seeded in a 24-well plate after nanoelectroporation (7300 cells/cm^2). Images were acquired every 20 minutes for 50 hours.

Movie S4

Phase holographic movie of cells transfected using nanostraws-Al, seeded in a 24-well plate after nanoelectroporation (7300 cells/cm^2). Images were acquired every 20 minutes for 50 hours.

Movie S5

Phase holographic movie of cells transfected using nanostraws-Al but without any plasmid in the cargo solution, called "Mock", and seeded in a 24-well plate after nanoelectroporation (7300 cells/cm^2). The cargo solution consisted of only MQ water. Images were acquired every 20 minutes for 50 hours**.**
